# NOP receptor pharmacological profile – A dynamic mass redistribution study

**DOI:** 10.1371/journal.pone.0203021

**Published:** 2018-08-30

**Authors:** Davide Malfacini, Katharina Simon, Claudio Trapella, Remo Guerrini, Nurulain T. Zaveri, Evi Kostenis, Girolamo Calo’

**Affiliations:** 1 Molecular, Cellular and Pharmacobiology Section, Institute for Pharmaceutical Biology, University of Bonn, Bonn, Germany; 2 Section of Pharmacology, Department of Medical Sciences, and National Institute of Neurosciences, University of Ferrara, Ferrara, Italy; 3 Department of Chemical and Pharmaceutical Sciences and LTTA, University of Ferrara, Ferrara, Italy; 4 Astraea Therapeutics, LLC, Mountain View, CA, United States of America; Universite de Rouen, FRANCE

## Abstract

The Nociceptin/Orphanin FQ (N/OFQ) peptide NOP receptor is coupled to pertussis toxin (PTX)-sensitive G proteins (G_i/o_) whose activation leads to the inhibition of both cAMP production and calcium channel activity, and to the stimulation of potassium currents. The label free dynamic mass redistribution (DMR) approach has been demonstrated useful for investigating the pharmacological profile of G protein-coupled receptors. Herein, we employ DMR technology to systematically characterize the pharmacology of a large panel of NOP receptor ligands. These are of peptide and non-peptide nature and display varying degrees of receptor efficacy, ranging from full agonism to pure antagonism. Using Chinese hamster ovary (CHO) cells expressing the human NOP receptor we provide rank orders of potency for full and partial agonists as well as apparent affinities for selective antagonists. We find the pharmacological profile of NOP receptor ligands to be similar but not identical to values reported in the literature using canonical assays for G_i/o_-coupled receptors. Our data demonstrate that holistic label-free DMR detection can be successfully used to investigate the pharmacology of the NOP receptor and to characterize the cellular effects of novel NOP receptor ligands.

## Introduction

Nociceptin/Orphanin FQ (N/OFQ) is a 17 amino-acid (FGGFTGARKSARKLANQ) neuropeptide that binds with high affinity to the N/OFQ peptide (NOP) receptor [1, 2]. The NOP receptor mainly couples to pertussis toxin (PTX)-sensitive G proteins (G_i/o_) whose activation leads to lowering of cAMP levels and inhibition of calcium channels, but also to the stimulation of potassium currents [3]. Its pharmacology has been classically studied in vitro with bioassays such as the electrically stimulated mouse vas deferens, and biochemical assays based on [^35^S]GTPγS binding and inhibition of forskolin-stimulated cAMP production. More recently, bioluminescence resonance energy transfer (BRET) based assays allowed the investigation of NOP/G protein and NOP/β-arrestin interactions demonstrating that several synthetic agonists are biased toward activation of G protein signaling over β-arrestin recruitment [4, 5]. Moreover, our knowledge about the binding pocket of the NOP receptor has been broadened substantially by the availability of the crystal structure of the NOP receptor in complex with different antagonists [6, 7]. The identification of several NOP receptor selective ligands [3, 8, 9] made it possible to test the in vivo consequences of selective stimulation or blockage of the NOP receptor. Complementary information has been collected using genetically modified animals such as mice [10] and rats [11] deficient in expression of the NOP receptor or the N/OFQ peptide precursor [12], and mice expressing a NOP-eGFP fusion protein from the native NOP receptor locus [13]. Pharmacological and genetic studies demonstrated the involvement of the N/OFQ-NOP receptor system in the control of different biological functions including pain, mood and anxiety, food intake, learning and memory, locomotion, drug abuse, cough and micturition reflexes, cardiovascular homeostasis, intestinal motility and immune responses [3, 14, 15].

NOP is a G protein-coupled receptor (GPCR), GPCRs are macromolecules belonging to the largest family of membrane proteins in the human genome. They are involved in the control of virtually all physiological processes and represent one of the main targets for prescribed medicines, in fact about 36% of all therapeutics mediate their effects through GPCRs [16]. The development of GPCR research in physiology and pharmacology led to a significant expansion of both available knowledge and methods for investigating these receptors [17–20]. The continuous acceleration in knowledge acquisition on GPCR conformational complexity (e.g. X-ray and CryoEM near atomic resolution structures) and how different ligands perturbate receptor signaling cascades (i.e. biased agonism), might increase the challenge in translating the effects elicited by receptor ligands from the medicinal chemistry to the biological level [21]. For this reason, the use of phenotypic biosensor technology platforms capable to measure whole cell integrated responses might provide a new angle towards detection and differentiation of promising GPCR ligands.

Such methods, rather than focusing at single readout assays (e.g. GTP/GDP exchange, second messengers’ levels modulation, protein-protein interaction, protein phosphorylation, etc.) make it possible to obtain a more global view of receptor-dependent cellular perturbations. The mostly used, are based on special biosensors (electron-conducting or light-diffracting plates) that allow translation of the receptor-dependent holistic cellular response to physical parameters such as variations in impedance or modulations of wavelength shift of an incident light in real time [22]. These assays are used in laboratories from both industry and academia and may be advantageous for identifying novel molecular entities with favorable in vitro profiles before translation to in vivo investigations. This is in part due to the possibility to test drug candidates non-invasively in several types of cellular backgrounds, including primary cell cultures.

The dynamic mass redistribution (DMR) assay is based on an optical biosensor technology, and was recently developed to monitor receptor signaling responses including those mediated by GPCRs (for details on the method see [23, 24]). It has already been applied to study the pharmacological properties of new ligands acting at various GPCRs such as the urotensin-II [25], β2 adrenergic [26, 27], muscarinic M3 [28], purinergic P2Y [29], formyl peptide [30], and protease activated [31, 32] receptors. Classical opioid receptors, the mu [33], kappa and delta receptors [34], were also evaluated with DMR. No data are yet available for the NOP receptor. Thus, in the present study we performed a systematic pharmacological characterization of the NOP receptor using a label-free optical DMR-based biosensor, cells expressing the human NOP receptor, and a large panel of NOP ligands with a wide spectrum of pharmacological activities.

## Materials and methods

### Drugs and reagents

The peptides N/OFQ, N/OFQ(1–13)-NH_2_, UFP-112, UFP-101, [F/G]N/OFQ(1–13)-NH_2_, [Nphe^1^]N/OFQ(1–13)-NH_2_, [Arg^14^,Lys^15^]N/OFQ, Ac-RYYRIK-NH_2_, and PWT2-N/OFQ were synthesized in house following previously described procedures [35, 36]. The non-peptide molecules Ro 65–6570, C-24, and J-113397 were synthesized in our laboratories. SB-612111 and naloxone were from Tocris bioscience (Bristol, UK). AT-090 and AT-127 were provided by N Zaveri (Astraea Therapeutics, Mountain View, USA). Pertussis toxin was from Sigma (Taufkirchen, DE). Hanks balanced salt solution (HBSS) was from Invitrogen (Darmstadt, DE), HEPES was from Applichem (Darmstadt, DE). All tissue culture media and supplements were from Invitrogen (Darmstadt, DE) and were of the highest purity available. Concentrated solutions of ligands were made in ultrapure water or dimethyl sulfoxide and kept at—20°C until use.

### Cells

Chinese Hamster Ovary (CHO) cells stably expressing the human NOP receptor (CHO_NOP_) were kindly provided by D.G. Lambert (University of Leicester, UK). CHO_delta_ were supplied by E Varga (The University of Arizona, USA), CHO_mu_ and CHO_kappa_ were both provided by L Toll (Torrey Pines Institute for Molecular Studies, Port St. Lucie, USA), CHO cells were used as control. Cells were cultured in Dulbecco's Modified Eagle Medium: Nutrient Mixture F-12 (DMEM/F12) supplemented with 10% (v/v) Fetal Calf Serum (FCS), 100 U/ml penicillin, 100 μg/ml streptomycin, 2 mM L-glutamine, 15 mM HEPES. The medium was supplemented with 400 μg/ml G418 to maintain expression.

### Experimental protocol

For DMR measurements the label-free EnSight Multimode Plate Reader (Perkin Elmer, MA, US) was used. Cells were seeded at 12,000 cells/well onto fibronectin-coated 384 well DMR microplates and cultured for 20 h to obtain confluent monolayers. Cells were starved in assay buffer (Hank’s Balanced Salt Solution (HBSS) with 20 mM HEPES, 0.01% Bovine Serum Albumin (BSA) fraction V) for 1 hr prior the addition of compounds. Serial dilutions were made in the assay buffer. After reading baseline, compounds were added using a semiautomatic liquid handler Selma (Analytik Jena AG, Jena, DE). Online additions of 10 μL compounds were carried out in a volume of 30 μl/well. Antagonists were incubated 30 min before agonist injection, then DMR changes were recorded for 3000 sec. Agonists responses represented in traces were described as picometer (pm) shifts over time (sec) following baseline normalization. Maximum picometers (pm) modification (Peak) and area under the curve (AUC) were used to generate concentration response curves. All the experiments were carried out at 37°C. For a detailed description of the methods see [24] and [37]

### Data analysis

All the data were elaborated using Graph Pad Prism 6.0 (La Jolla, CA, US). Concentration response curves were fitted by log logistic four parameter equation. Data are expressed as mean + or ± sem of n experiments and were analyzed statistically using one-way analysis of variance followed by Dunnett’s test for multiple comparisons. Agonist potencies are given as pEC_50_ i.e. the negative logarithm to base ten of the molar concentration of an agonist that produces half of the maximal effect. Agonist maximal effect, i.e. the maximal effect that an agonist can elicit in a given preparation under particular experimental conditions, has been also expressed as intrinsic activity (α) by dividing the Emax of the agonist under study by that elicited by the reference full agonist (N/OFQ) in parallel experiments. Antagonists were assayed at single concentrations against the concentration-response curve to the agonist and their potencies expressed as pK_B_ according to the following equation: pK_B_ = log(CR-1)-log[A], where CR is the ratio between agonist potency (expressed as EC_50_) in the presence and absence of antagonist and [A] is the molar concentration of antagonist. K_B_ refers to the equilibrium dissociation constant of a ligand determined by means of a functional assay [38]. In a separate series of experiments SB-612111 was tested using the classical Schild protocol.

## Results

**Fig 1:**
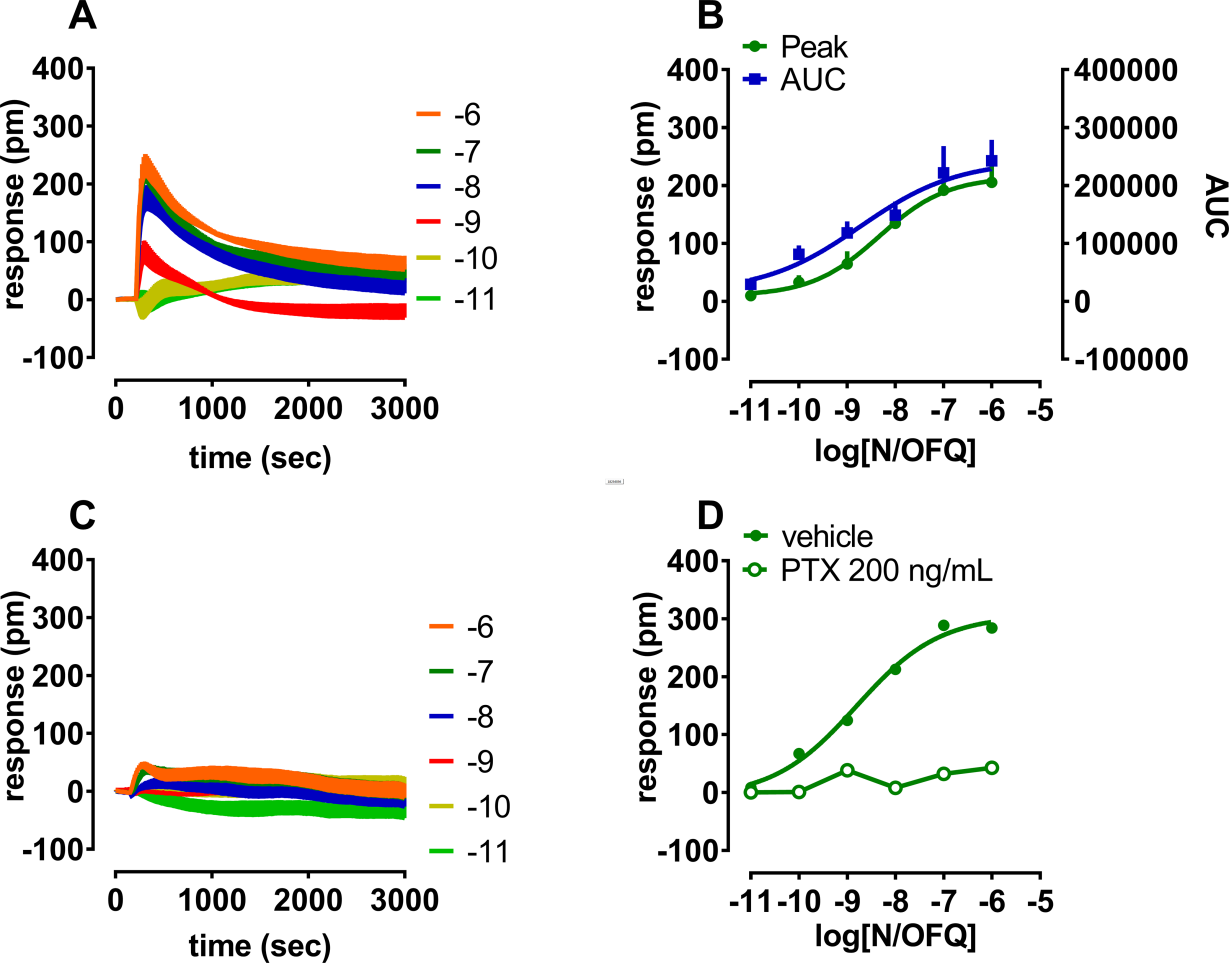
**Concentration response curve to N/OFQ (10 pM– 1 μM) in the absence (panels A and B) and presence of 200 ng/mL PTX (panels C and D)**. Baseline corrected DMR representative tracings are shown in panels A and C and concentration response curves in panels B and D. Sigmoidal curves to N/OFQ computed as peak and AUC are shown as mean + sem of at least 3 experiments performed in triplicate (panel B).

Cellular effects of the endogenous peptide N/OFQ were measured over time at increasing concentrations applying the DMR technology; at 1 μM the signal-to-noise ratio calculated was in the range 4–4.5. N/OFQ effects were thereafter computed in sigmoidal curves as peaks and areas under the curve (AUC); similar values of potency were obtained by fitting the two parameters (pEC_50_ 8.33 and 8.73, respectively) (Fig 1A and 1B). For simplicity concentration response curves to NOP agonists are later presented as DMR peaks. To confirm the prevalent G_i/o_ nature of the N/OFQ-stimulated DMR responses, N/OFQ effects were measured after 20 h pretreatment with 200 ng/mL PTX. The G_i/o_ selective ADP-ribosylator largely diminished the N/OFQ DMR signal confirming the signaling preferences of the NOP receptor (Fig 1C and 1D). Importantly, N/OFQ was completely inactive when tested in wild type CHO cells (Table 1).

**Table 1:** DMR responses to high concentrations of ligands in CHO and CHO_NOP_ cells.

	CHO	CHO_NOP_
	(pm ± sem)	(pm± sem)
N/OFQ	23±9	205±29*
N/OFQ(1–13)NH_2_	21±8	267±17*
[Arg^14^,Lys^15^]N/OFQ	40±20	271±16*
UFP-112	25±7	248±38*
PWT2-N/OFQ 0.1 μM	24±5	198±55*
PWT2-N/OFQ 1 μM	467±23*	~ 350*
[Nphe^1^]N/OFQ(1–13)NH_2_	20±6	180±21*
[F/G]N/OFQ(1–13)NH_2_	24±2	200±17*
UFP-101	25±6	~ 40
Ac-RYYRIK-NH_2_	17±8	182±11*
Ro 65–6570	23±8	210±19*
AT-090	21±3	252±15*
AT-127	20±4	191±5*
SB-612111	26±3	-10±9
J-113397	17±8	5±16
C-35	16±4	-9±14
C-24	21±5	-13±15
Dermorphin	35±12	25±20
DPDPE	21±5	12±13
Dynorphin A	7±4	32±14
Naloxone	23±5	15±12
FSK	-172±23*	-164±15*
ATP	177±15*	185±12*
Buffer	18±10	12±22

PWT2-N/OFQ was tested at 0.1 and 1 **μ**M, FSK and ATP at 100 **μ**M, all the other compounds at 1 **μ**M.

*p < 0.05 vs buffer according to one-way ANOVA followed by the Dunnett’s test for multiple comparisons.

### DMR effects of NOP full and partial agonists

**Fig 2:**
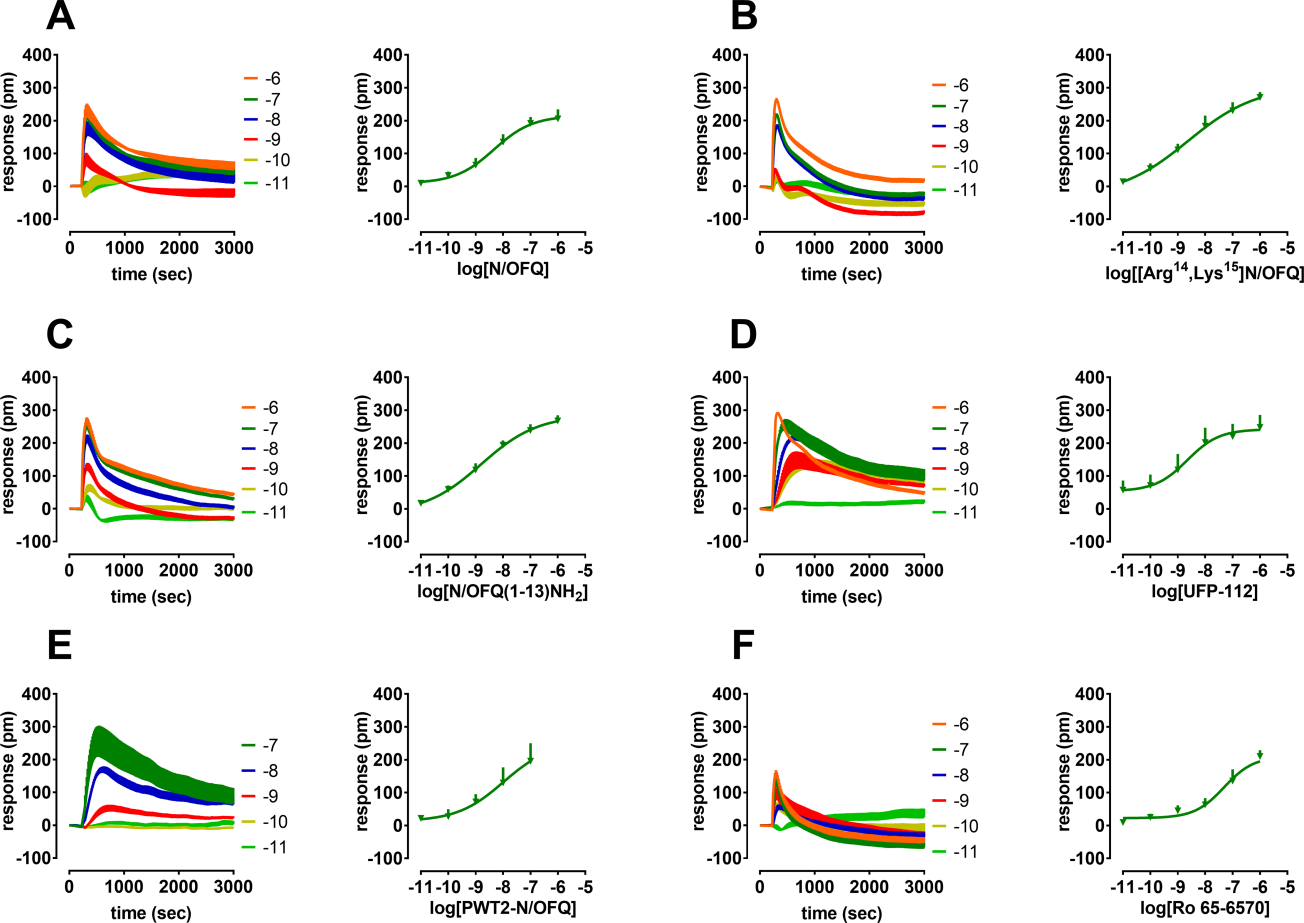
**Concentration response curve to N/OFQ (panel A), [Arg^14^,Lys^15^]N/OFQ (panel B), N/OFQ(1–13)-NH_2_ (panel C), UFP-112 (panel D), PWT2-N/OFQ (panel E), and Ro 65–6570 (panel F)**. Representative raw DMR tracings are represented on the left of each panel and average sigmoidal curves on the right. Data are mean + sem of at least 3 separate experiments performed in triplicate.

**Fig 3:**
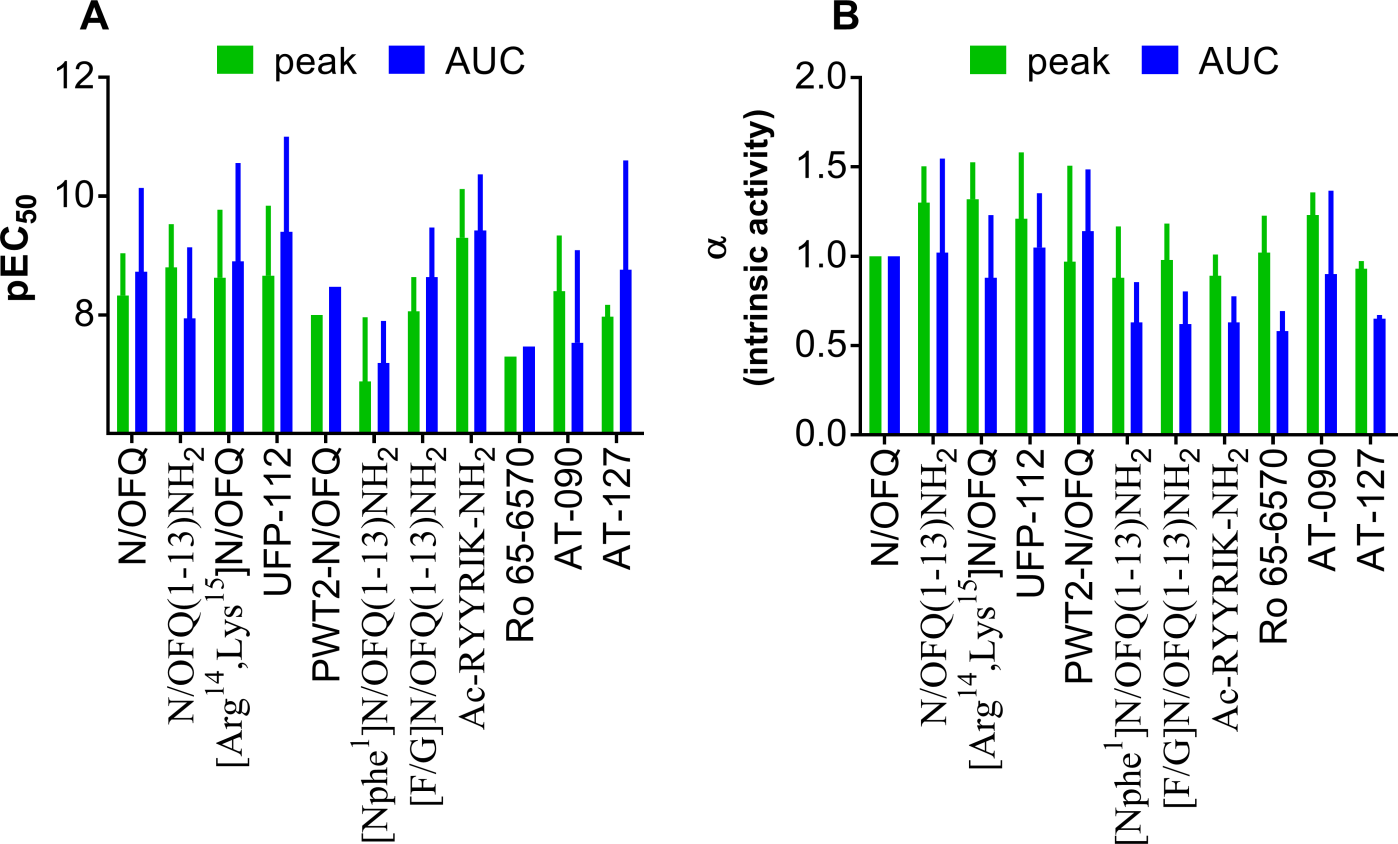
**Comparison of potencies (pEC_50_ + CL_95%_, panel A) and maximal effects (α+ SD, panel B) of NOP receptor agonists obtained by computing maximal DMR peaks or areas under the curve (AUC)**.

The rank order of potency of selective agonists was determined in the DMR assay by studying a panel of NOP ligands encompassing full and partial agonist activity together with the endogenous peptide N/OFQ (Fig 2A). N/OFQ(1–13)-NH_2_, a peptide constituted by the minimal sequence maintaining the same activity as N/OFQ, mimicked the stimulatory effects of the endogenous peptide with similar potency (pEC_50_ 8.80) and efficacy (Emax 267) (Fig 2C). The N/OFQ derivatives [Arg^14^,Lys^15^]N/OFQ and UFP-112 also displayed similar effects as N/OFQ showing comparable high potency (pEC_50_ 8.63 and 8.66) and maximal effects (Emax 271 and 248) (Fig 2B and 2D). The effects of the recently developed N/OFQ tetrabranched peptide PWT2-N/OFQ were tested up to 0.1 μM since at 1 μM this compound was active in wild type CHO cells (Table 1) and potency and maximal effects estimated were similar to that of N/OFQ (pEC_50_ ~ 8, Emax 198) (Fig 2E). The effects of the tetrabranched peptide appeared, in 3 out of 6 experiments, longer lasting than those elicited by N/OFQ. Of note, the Emax of PWT2-N/OFQ calculated as AUC were not significantly, yet higher than those of N/OFQ (Fig 3B). Ro 65–6570, one of the most commonly used non-peptide NOP agonists, produced a concentration-dependent increase in the DMR signal without reaching the stimulation plateau; the application of higher concentrations of compound was not possible since Ro 65–6570 exhibited a DMR response in wild type CHO cells when tested at 10 μM. The concentration response curve for Ro 65–6570 was constrained to the estimated maximal effects and a value of potency of ~ 7.3 was calculated (Fig 2F).

**Fig 4:**
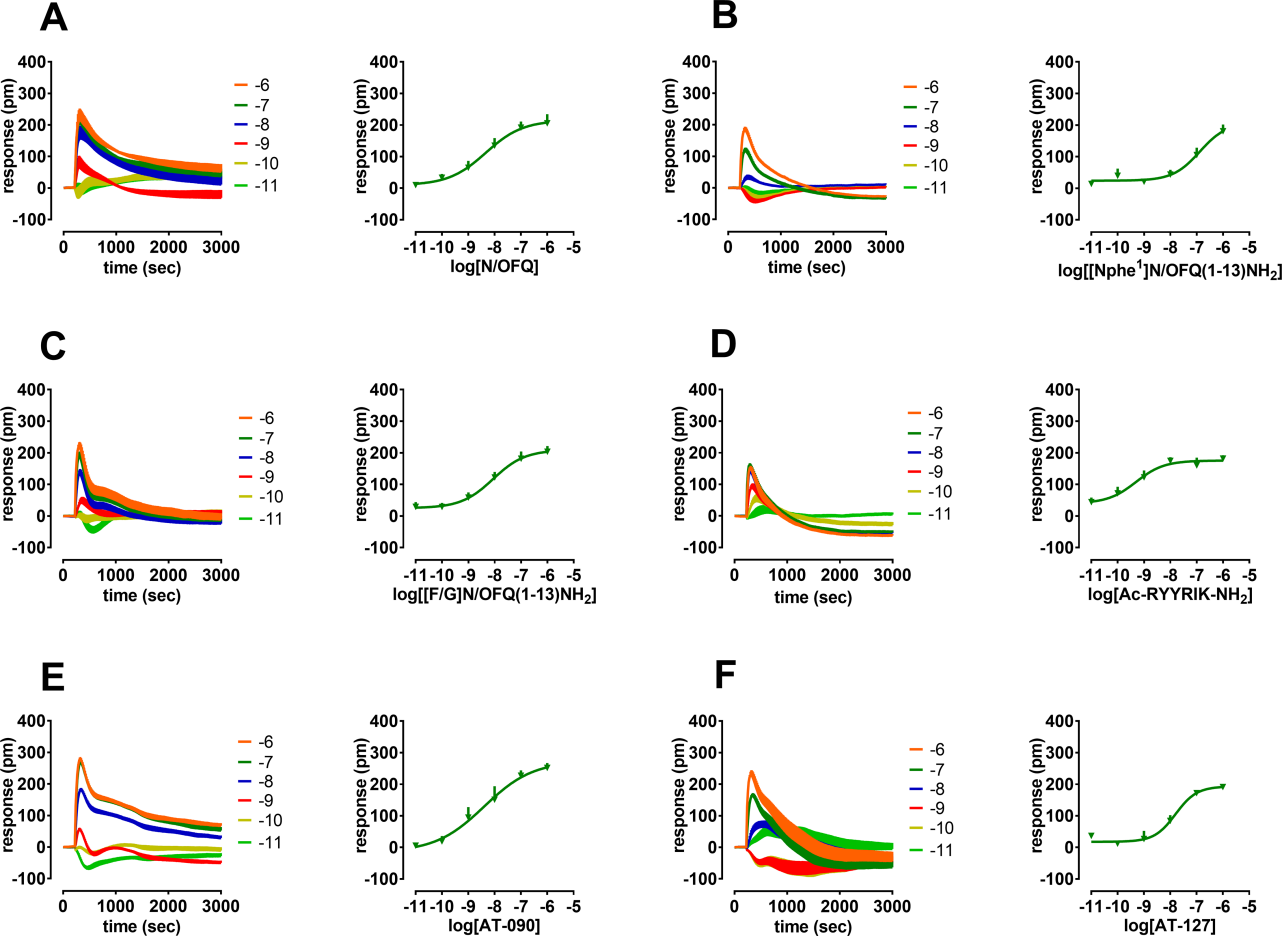
**Concentration response curve to N/OFQ (panel A), [Nphe^1^]N/OFQ (panel B), [F/G]N/OFQ(1–13)-NH_2_ (panel C), Ac-RYYRIK-NH_2_ (panel D), AT-090 (panel E), and AT-127 (panel F)**. Representative raw DMR tracings are represented on the left of each panel and average sigmoidal curves on the right. Data are mean + sem of at least 3 separate experiments performed in triplicate.

In Fig 4 DMR traces of NOP partial agonists are displayed and computed as concentration response curves in comparison with N/OFQ (Fig 4A). The peptide [Nphe^1^]N/OFQ(1–13)-NH_2_ stimulated the NOP receptor mimicking the effects of N/OFQ but with lower potency (~30-fold) and maximal effects (Emax 180) (Fig 4B). The first N/OFQ related peptide showing reduced efficacy, [F/G]N/OFQ(1–13)-NH_2_, concentration dependently stimulated DMR effects with comparable potency (pEC_50_ 8.06) as the endogenous peptide (Fig 3C). The hexapeptide Ac-RYYRIK-NH_2_ evoked a concentration dependent stimulation of the NOP receptor with estimated potency approximately 10-fold higher than N/OFQ and similar maximal effects (Emax 182) (Fig 4D). The recently characterized non-peptide agonists AT-090 and AT-127 showed high potency (pEC_50_ 8.40 and 7.97) and maximal effects (Emax 252 and 191) (Fig 4E and 4F).

**Fig 5:**
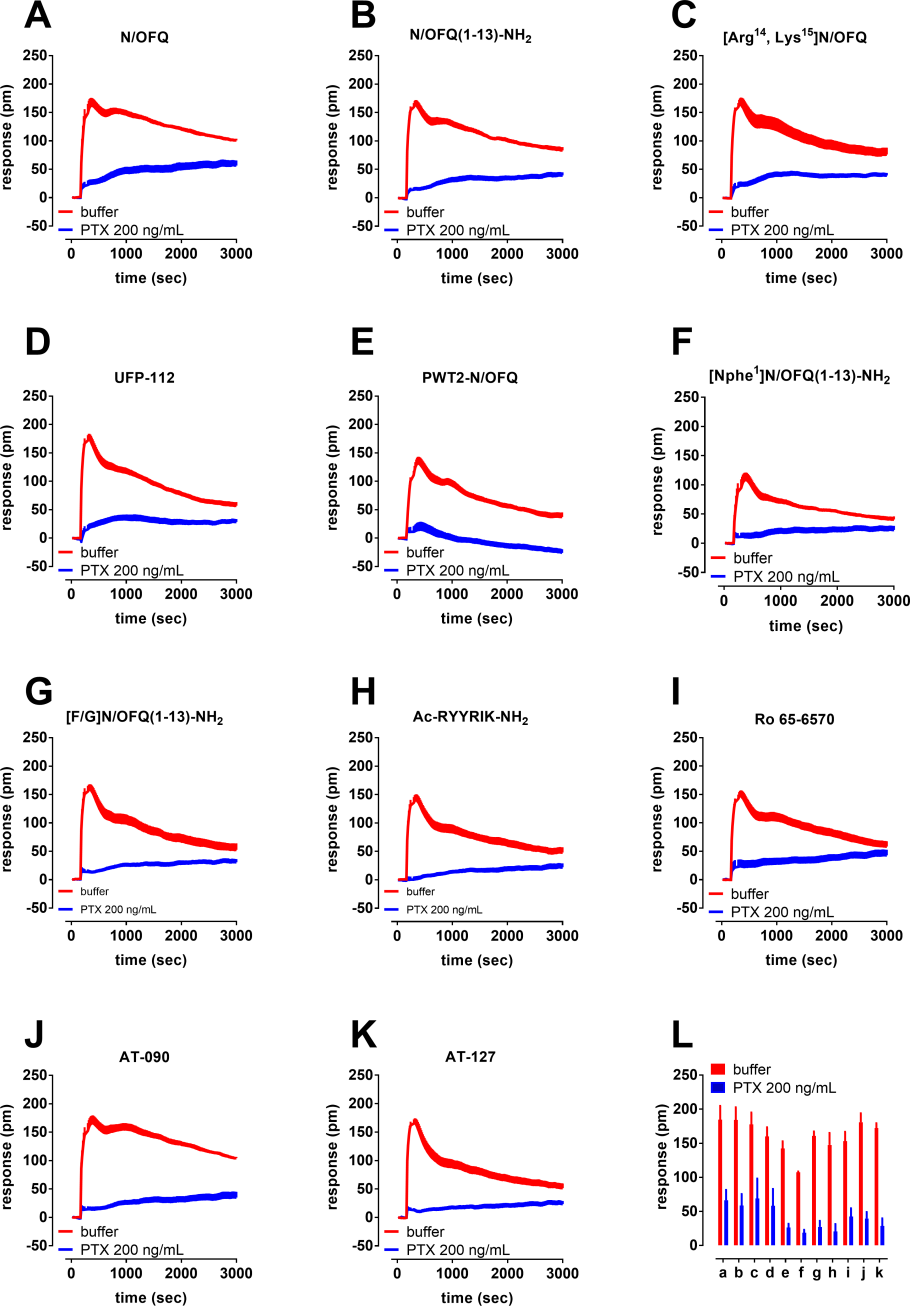
Representative DMR traces for NOP receptor agonists (panels A-K) tested at 1 μM or at 0.1 μM (PWT2-N/OFQ, panel E), in the absence and presence of 200 ng/mL PTX. The effects of the same compounds are reported as DMR peaks in the absence and in the presence of 200 ng/mL PTX in panel L. Data in panel A-K are mean + sem of a single experiment performed in triplicate. Data in panel L are mean + SD of 3 separate experiments performed in triplicate.

In a separate series of experiments, the nature of the NOP-DMR signal elicited by full and partial agonists was investigated by testing the ligands at the single concentration of 1 μM (with the exception of PWT2-N/OFQ that was tested at 0.1 μM) in cells treated with PTX (Fig 5). The effects of all compounds were largely blunted by toxin pretreatment with residual DMR signal ranging from 15 to 40% of the control response (Fig 5L).

Comparison of the effects of high concentrations of ligands in wild type CHO and CHO_NOP_ cells is shown in Table 1.

### DMR effects of antagonists at the NOP and classical opioid receptors

**Fig 6:**
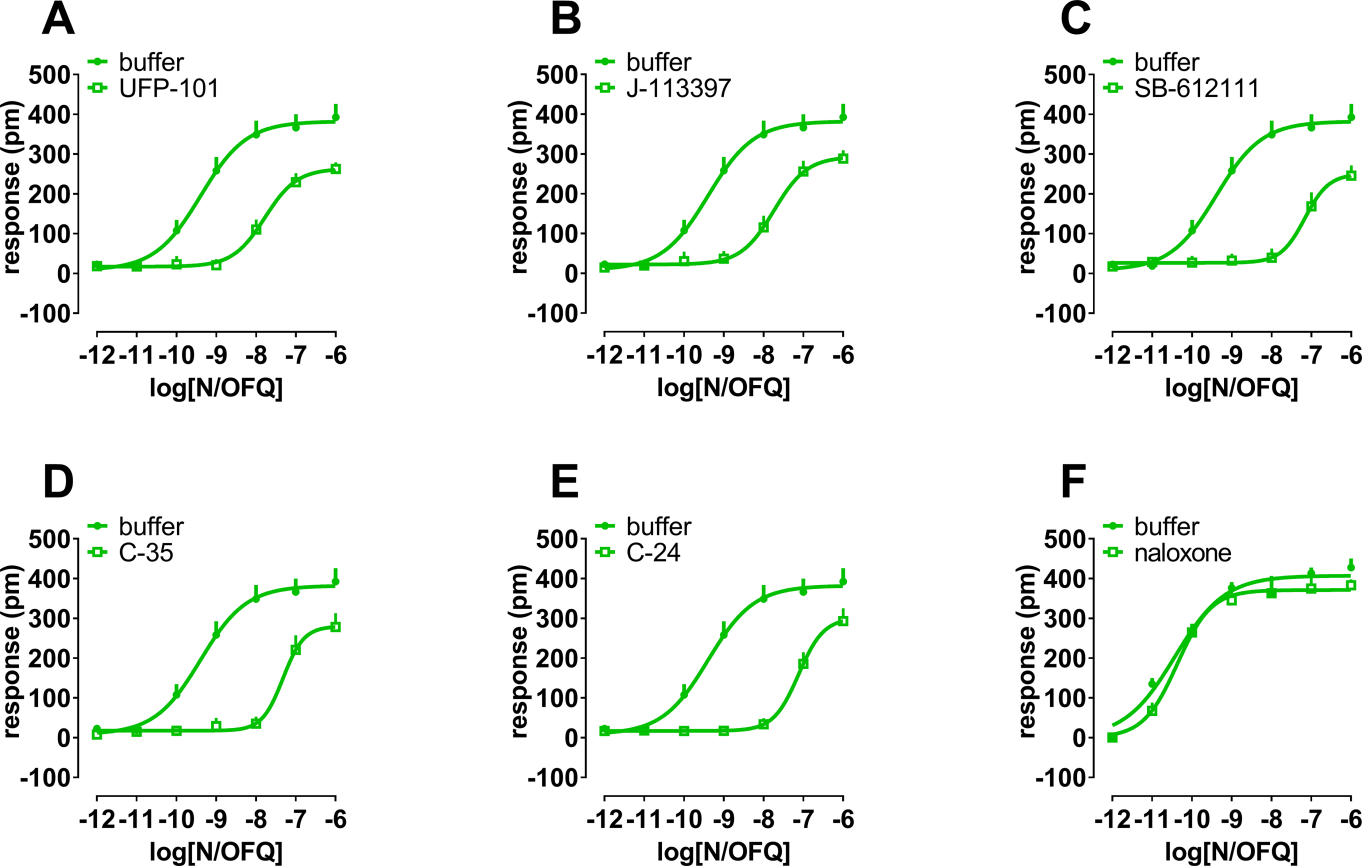
**Concentration response curve to N/OFQ in the absence and presence of 1 μM UFP-101 (panel A), J-113397 (panel B), SB-612111 (panel C), C-35 (panel D), C-24 (panel E), and naloxone (panel F)**. Data are mean + sem of at least 3 separate experiments performed in triplicate.

**Fig 7:**
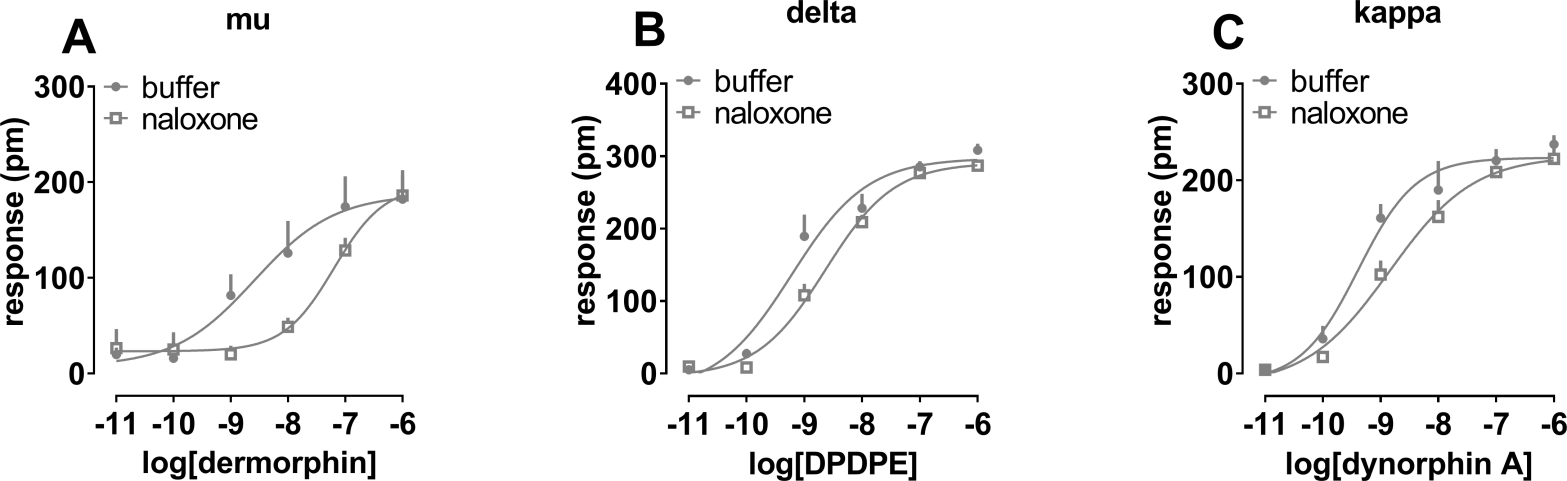
**Concentration response curve to dermorphin (panel A), DPDPE (panel B), and dynorphin A (panel C) in the absence and presence of 100 nM naloxone**. Data are mean + sem of 3 separate experiments performed in triplicate.

Finally, the effects of the classical opioid receptor antagonist naloxone, and the NOP receptor antagonists UFP-101, J-113397, SB-612111, C-35, and C-24 were tested (at fixed concentrations of 1 μM) against the concentration response curve to N/OFQ. These compounds did not produce any effect per se in CHO_NOP_ cells with the exception of UFP-101 which elicited a stimulatory effect approximately corresponding to 20% of the maximal effects of N/OFQ (S1 Fig). All the compounds elicited a rightward shift of the concentration response curve to N/OFQ (Fig 6) with estimated pK_B_ values of 7.60, 7.65, 8.25, 8.07, and 8.30 for UFP-101, J-113397, SB-612111, C-35, and C-24, respectively. The antagonists caused a slight depression of the N/OFQ maximal effects at the concentrations tested. On the contrary naloxone did not modify the concentration response curve to N/OFQ. The opioid antagonist was also tested in cells expressing the classical opioid receptors against the standard agonists dermorphin (pEC_50_ 8.59), DPDPE (pEC_50_ 9.22), and dynorphin A (pEC_50_ 9.39) for mu, delta, and kappa receptors, respectively. Naloxone shifted to the right the concentration response curves to opioid agonists without affecting their maximal effects with estimated pK_B_ values of 8.37 at mu, 7.53, at delta, and 7.35 at kappa opioid receptor (Fig 7).

**Fig 8:**
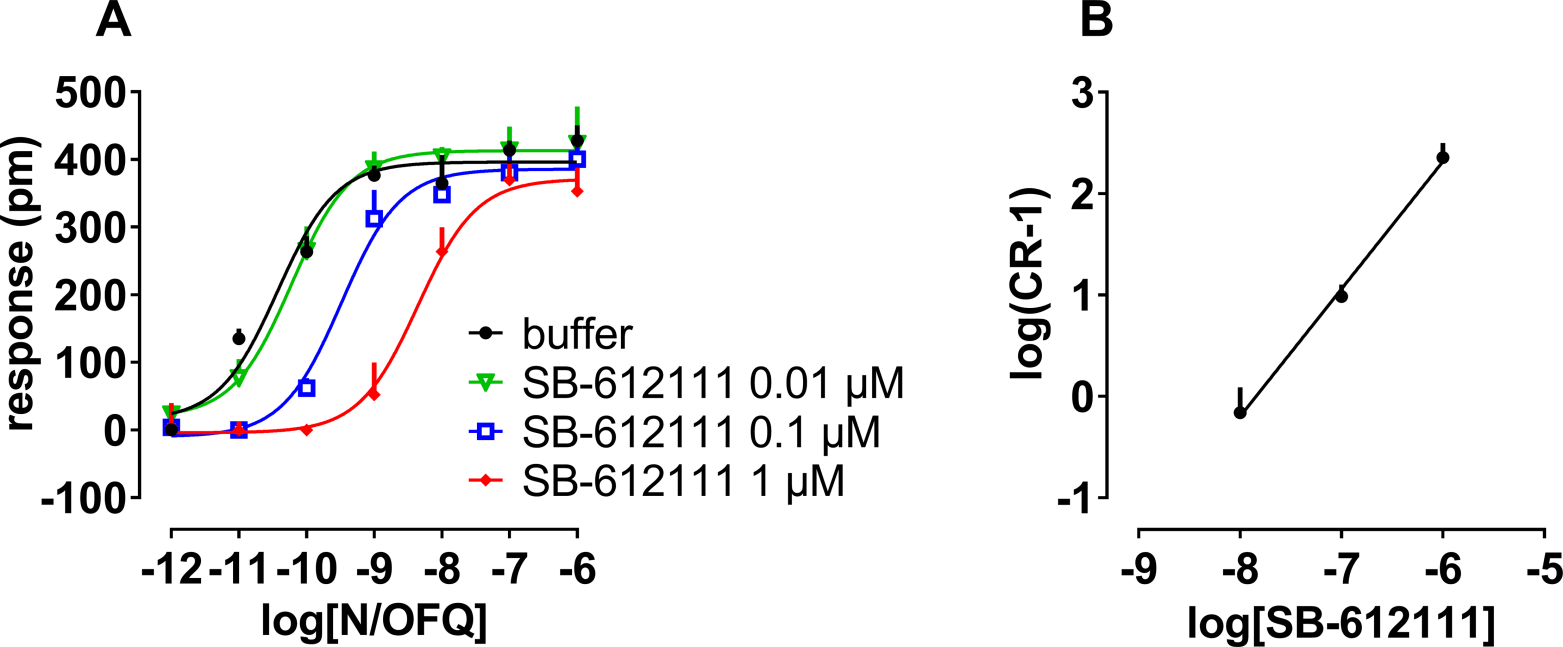
Concentration-response curves to N/OFQ in absence and presence of increasing concentrations (10 nM– 1 μM) of SB-612111 (panel A). The corresponding Schild plot is shown in panel B. Data are mean + sem of 3 separate experiments performed in triplicate.

The antagonist nature of SB-612111 was further characterized by the classical Schild protocol by challenging the concentration-response curve to N/OFQ with increasing concentrations of the antagonist. SB-612111 rightward shifted the agonist curve without significantly affecting its maximal effects; a pA_2_ of 7.84 and a slope value close to 1 were obtained from the relative Schild plot (Fig 8).

**Fig 9:**
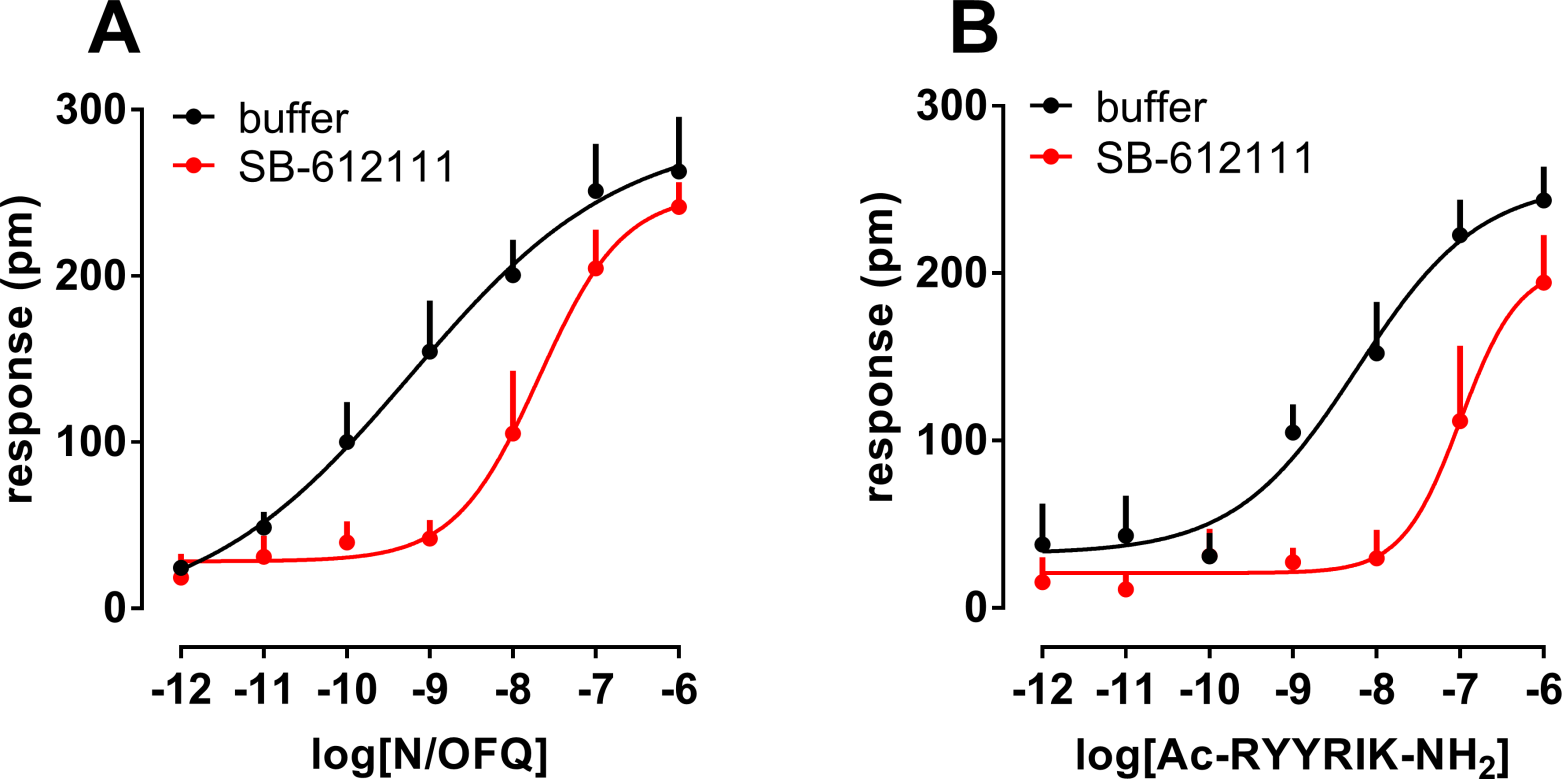
**Concentration response curve to N/OFQ (panel A) and Ac-RYYRIK-NH_2_ (panel B) in the absence and presence of 1 μM SB-612111**. Data are mean + sem of at least 3 separate experiments performed in triplicate.

In a separate series of experiments, the effects of increasing concentrations of N/OFQ and Ac-RYYRIK-NH_2_ were studied in the absence and presence of SB-612111 (1 μM), The antagonist produced a similar dextral displacement of the concentration response curve to N/OFQ and Ac-RYYRIK-NH_2_ and the calculated pK_B_ values were 7.53 and 7.21, respectively (Fig 9).

Pharmacological parameters of the NOP ligands investigated in the present study have been schematically summarized in Table 2.

**Table 2:** Agonist potencies (pEC_50_) and intrinsic activity (α), and antagonist potencies (pK_B_) of the compounds tested in the CHO_NOP_ cell DMR assay.

	pEC_50_	α	pK_B_
N/OFQ	8.33 (7.63–9.04)	1.00	
N/OFQ(1–13)NH_2_	8.80 (8.07–9.53)	1.30	
[Arg^14^,Lys^15^]N/OFQ	8.63 (7.48–9.77)	1.32	
UFP-112	8.66 (7.47–9.84)	1.21	
PWT2-N/OFQ	~8.00	0.97	
[Nphe^1^]N/OFQ(1–13)NH_2_	6.88 (5.80–7.96)	0.88	
[F/G]N/OFQ(1–13)NH_2_	8.06 (7.48–8.64)	0.98	
Ac-RYYRIK-NH_2_	9.30 (8.49–10.12)	0.89	
Ro 65–6570	~7.30	1.02	
AT-090	8.40 (7.46–9.34)	1.23	
AT-127	7.97 (7.42–8.17)	0.93	
UFP-101	crc incomplete		7.60 (7.44–7.76)
SB-612111	inactive		8.25 (7.98–8.53)
J-113397	inactive		7.65 (7.45–7.85)
C-35	inactive		8.07 (7.90–8.24)
C-24	inactive		8.30 (8.04–8.57)

Inactive means that up to 1 μM the compound did not promote any DMR response.

## Discussion

In the present study we have used the DMR technique that allows an integrated non-invasive measurement of cellular function, to investigate the pharmacological profile of the human NOP receptor in recombinant cells. A panel of peptide and non-peptide selective NOP ligands with a wide range of potency and efficacy, from full agonism to pure antagonism were studied. PTX experiments revealed that NOP signaling in CHO cells is largely yet not exclusively due to G_i/o_ coupling. The DMR pharmacological profile of the NOP receptor in terms of rank order of potency of full and partial agonists and apparent affinity of selective antagonists is similar although not identical to that reported in the literature using standard assays for G_i/o_ coupled receptors.

N/OFQ stimulated the DMR response in CHO_NOP_ cells but not in CHO cells demonstrating that this signal exclusively derives from the interaction of N/OFQ with the NOP receptor protein. The same is true for all the agonists evaluated since we selected their concentration range based on lack of DMR signal in CHO cells. Regarding the transduction pathway involved in the NOP dependent DMR signal, pretreating the cells with PTX largely inhibited the DMR signal elicited by N/OFQ. Consistently, the DMR response to all NOP agonists were depleted by toxin treatment, to a larger extent for partial than full agonists. This result demonstrated that in CHO cells the DMR signal is mainly, albeit not completely, due to NOP coupling with G proteins of the G_i/o_ family. PTX treatment is known to block most of the inhibitory Gα proteins through ADP-ribosylation of a Cys351 residue [39]. Importantly, previous reports described that the NOP receptor is able to couple to PTX-insensitive G proteins such as G_z_ and G_16_ [40], but also to G_12_ and G_14_ [41]. However the PTX resistant DMR signal elicited by N/OFQ as well as NOP agonists in CHO cells is too small to investigate further. In the future we will look for cells (possibly expressing the native NOP receptor) in which the G_i/o_ independent component of the DMR signal in response to N/OFQ is large enough to be investigated in deconvolution studies. Moreover, despite mechanistic details of arrestin catalytic activation are now being described [42], the lack of functional G proteins does not allow for arrestin-mediated signaling. [43]. Therefore these deconvolution studies will be validated by employing CRISPR/Cas9-edited cells lacking in turn G proteins or arrestins.

The DMR response to NOP activation by a series of NOP full agonists including the peptides N/OFQ(1–13)-NH_2_ [36], [Arg^14^, Lys^15^]N/OFQ [44], and UFP-112 [45], the N/OFQ tetrabranched derivative PWT2-N/OFQ [46], and the non-peptide molecule Ro 65–6570 [47] was investigated in the first series of experiments. These compounds mimicked the stimulatory effects of N/OFQ with similar maximal effects. Thus, in line with the original findings these molecules behave as full agonists at the NOP receptor. As far as agonist potency is concerned the following rank order has been measured:

$$N/\mathrm{OFQ}\left(1‑13\right){‑\mathrm{NH}}_{2}\geq \left[{\mathrm{Arg}}^{14}{,\mathrm{Lys}}^{15}\right]N/\mathrm{OFQ}=\mathrm{UFP}‑112>N/\mathrm{OFQ}\geq \mathrm{PWT}2‑N/\mathrm{OFQ}>\mathrm{Ro}\;65‑6570.$$

This is in general in line with literature reports (see Table 2 of [3] that summarizes the action of these compounds in various assays/preparations at human recombinant and rodent native NOP receptors). However, there are some aspects that deserve attention. PWT2-N/OFQ has been reported to be more potent than N/OFQ in receptor binding, stimulated GTPγS binding, bioassay experiments [46] and more recently in a BRET based assay measuring NOP/G protein interaction [5]. In the present study the potency of PWT2-N/OFQ could not be precisely estimated since the compound produced off target effects at micromolar concentrations. This observation implies a certain loss of selectivity due to application of the PWT chemical modification to the N/OFQ peptide sequence and this is in line with bioassay studies in NOP knockout tissues where off target effects were observed with PWT2-N/OFQ but not N/OFQ [46]. PWT2-N/OFQ behavior in cells expressing the NOP receptor is interesting. In fact, in three out of six experiments, the tetrapeptide displayed a DMR response over time more sustained than N/OFQ. This is reminiscent of the behavior of this ligand in bioassay experiments where PWT2-N/OFQ elicited slow developing, long lasting and wash resistant effects [35]. Similar findings were obtained with different PWT peptides [48]. This feature, i.e. longer-lasting binding to the receptor, has been interpreted considering the mechanisms proposed to explain the mode of action of multivalent ligands that include receptor clustering, cooperative binding, rebinding and subsite binding [49]. This aspect of PWT2-N/OFQ action can be important since long lasting receptor binding contributes to prolongation of the duration of action (and eventually an increase in effect) *in vivo* [50]. As a matter of fact, in vivo PWT2-N/OFQ mimicked the spinal antinociceptive effects of the N/OFQ in models of nociceptive and neuropathic pain in mice and in non-human primates displaying approximately 40-fold higher potency and a remarkably prolonged duration of action [51]. Moreover when injected supraspinally in mice PWT2-N/OFQ stimulated food intake being 40 fold more potent than N/OFQ and eliciting larger effects [35].

A more detailed comparison of the present data with the literature shows that highly potent peptide agonists such as [Arg^14^, Lys^15^]N/OFQ and particularly UFP-112 were 10 to 30 fold more potent than N/OFQ in stimulated GTPγS binding and NOP/G protein interaction experiments while in the DMR assay this difference in potency is limited to 2 fold. Possibly differences in signal amplification, receptor desensitization and internalization between the assays may account for these differences.

In the second set of experiments a series of compounds with known partial agonist activity at the NOP receptor, the peptides [F/G]N/OFQ(1–13)-NH_2_ [52] and Ac-RYYRIK-NH_2_ [53] and the non peptides AT-090 and AT-127 [54], were evaluated. In the DMR assay all these compounds produced maximal effects that were not statistically different to those of N/OFQ. Similar results were obtained in calcium mobilization studies performed in cells co-expressing the NOP receptor and chimeric G proteins [54, 55]. On the contrary these same compounds consistently displayed significantly lower efficacy than N/OFQ in GTPγS binding and NOP/G protein interaction experiments [3, 5, 54]. As discussed in [56], this apparent discrepancy is probably due to the fact that the estimated efficacy of partial agonists strongly depends on the efficiency of the stimulus–response coupling which is different in the different assays. When the signal amplification phenomena make the efficiency of the stimulus–response coupling high, as in the case of DMR and calcium mobilization, ligand efficacy is overestimated. On the other hand, when there is little or no amplification and the efficiency of the stimulus-response coupling is low, as in the case of GTPγS binding and NOP/G protein interaction, ligand efficacy is underestimated. This phenomenon has been investigated in detail using a NOP-inducible expression system where the efficacy partial agonists could be manipulated to encompass full and partial agonism along with pure antagonism by changing the number of membrane receptors [57]. Importantly this does not happen only in recombinant systems but also when the receptor is investigated in a physiologically relevant environment. In fact [F/G]N/OFQ(1–13)-NH_2_ has been reported to act as a NOP antagonist in the electrically stimulated mouse vas deferent [52] and as a NOP full agonist in the mouse colon [58]. Interestingly, in vivo the compound acted as full agonist in the tail withdrawal assay [59], as partial agonist when measuring locomotor activity [60] and as a pure antagonist in the cardiovascular system, blocking N/OFQ-induced bradycardia and hypotension [61] in mice.

As far as potency of partial agonist is concerned the following rank order has been measured:

$${\mathrm{Ac}‑\mathrm{RYYRIK}‑\mathrm{NH}}_{2}>\mathrm{AT}‑090>\left[F/G\right]N/\mathrm{OFQ}\left(1‑13\right){‑\mathrm{NH}}_{2}=\mathrm{AT}‑127,$$

that perfectly matches previous results reported in the literature [3, 54].

In addition, evidence of negative DMR traces has been observed for some of the agonists tested in some but not all of the experiments carried out, e.g. Ac-RYYRIK-NH_2_ displayed, in some but not all of the experiments carried, a concentration dependent negative signal after 1000 sec with potency values determined at negative peaks being superimposable to those at positive peaks. The reasons for this action of Ac-RYYRIK-NH_2_ are unknown.

It is worthy of mention that previous photo-affinity labelling experiments demonstrated that the NOP binding pocket for Ac-RYYRIK-NH_2_ [62] and for N/OFQ [63] are distinct although overlapping, and this may favor the selection of different conformations and eventually coupling of the NOP receptor in response to these ligands. However, DMR responses to N/OFQ and Ac-RYYRIK-NH_2_ were equally sensitive to PTX and to the NOP selective antagonist SB-612111. These results exclude, at least under the present conditions, major differences in the way Ac-RYYRIK-NH_2_ activates the NOP receptor in comparison to the natural ligand N/OFQ.

Finally a panel of NOP antagonists including [Nphe^1^]N/OFQ(1–13)-NH_2_ [64], UFP-101 [65], J-113397 [66], SB-612111 [67], C-24 [68], and C-35 [69] were tested in DMR experiments per se and against the stimulatory effects elicited by N/OFQ. All non-peptide compounds did not modify per se the DMR baseline, while [Nphe^1^]N/OFQ(1–13)-NH_2_ and UFP-101 elicited a stimulatory action with maximal effects of 0.88 and 0.20 (N/OFQ = 1.00). A substantial body of evidence reviewed in [70] demonstrated the in vitro and in vivo NOP antagonist features of [Nphe^1^]N/OFQ(1–13)-NH_2_ and UFP-101. However there are also some limited results that suggest the elimination of ligand efficacy by the Phe^1^ / Nphe^1^ substitution might not be complete. In fact, sodium and GTP concentrations affected the potency of [Nphe^1^]N/OFQ(1–13)-NH_2_ in a manner similar to that of agonists (N/OFQ) but not of pure antagonists (J-113397). In electrophysiological experiments, C-24 or Trap-101 behaved as pure antagonists in control neurons and as inverse agonists in neurons microinjected with a NOP receptor coding plasmid. In contrast, UFP-101 acted as an antagonist in control cells while it displayed partial agonist behavior in transfected neurons [71]. Finally, it has been recently reported that both [Nphe^1^]N/OFQ(1–13)-NH_2_ and UFP-101 displayed some residual agonists activity (0.55 and 0.14, respectively) in a BRET NOP/G protein interaction assay [5]. The amount of agonist activity of [Nphe^1^]N/OFQ(1–13)-NH_2_ did not allow antagonist experiments to be performed with this compound while UFP-101, together with non peptide molecules, was further investigated for its ability to counteract N/OFQ stimulated DMR responses. All compounds produced a dextral displacement of the concentration response curve to N/OFQ with the following rank order of antagonist potency:

C‑24≥SB‑612111≥C‑35>J‑113397≥UFP‑101

which is in agreement with data in the literature [3]. Importantly, in line with a large body of evidence the action of N/OFQ at the NOP receptor was not antagonized by naloxone. The antagonist nature of SB-612111 has also been investigated using the classical Schild analysis confirming the competitive nature of this NOP receptor selective antagonist [5, 67, 72, 73]. Moreover, the same panel of NOP antagonists has been recently evaluated in parallel experiments performed with a BRET NOP/G protein interaction assay obtaining superimposable results. Interestingly, antagonist potency correlated with ability to induce receptor stability and crystallogenesis. Using this screening strategy, two structures of NOP in complex with candidate ligands SB-612111 and C-35 were solved [7] and compared to that previously obtained using C-24 [6].

Collectively the results obtained in this study demonstrated that the DMR assay can be successfully used to investigate the pharmacology of the NOP receptor, to characterize the effects of novel NOP receptor ligands, and to explore their signaling profile. In general this study confirms and extends previous findings (see studies quoted in the introduction section) demonstrating the usefulness of the DMR as an “integrative pharmacology” approach to be used to complement reductionist signaling pathway based approaches [74]. The potential value of the DMR assay goes far beyond its utility for basic pharmacology and drug screening investigations. In fact, the DMR assay can be used for investigating cell sensitivity to endogenous signals and drugs in cell lines expressing the native receptor or in primary cultured cells obtained from normal animals and models of pathology. In the longer-term, DMR studies can be performed comparing cellular responses in primary culture cells from normal subjects and from patients, from patients at different stages of disease and from patients treated with different drugs. These kinds of studies will contribute to the translational of medicine knowledge thus reducing the gap between discoveries in biomedical science and their safe and effective clinical application.

## Supporting information

S1 Fig **Concentration response curves to UFP-101 (panel A), J-113397 (panel B), SB-612111 (panel C), C-35 (panel D), C-24 (panel E), and naloxone (panel F)**. Representative traces were obtained from a representative experiment performed in triplicate.
(DOCX)
