# Supplementary figures and images for: NOP receptor pharmacological profile – A dynamic mass redistribution study
Source: PLoS One. 2018 Aug 30;13(8):e0203021. doi: 10.1371/journal.pone.0203021 (PMC6117024; doi:10.1371/journal.pone.0203021)

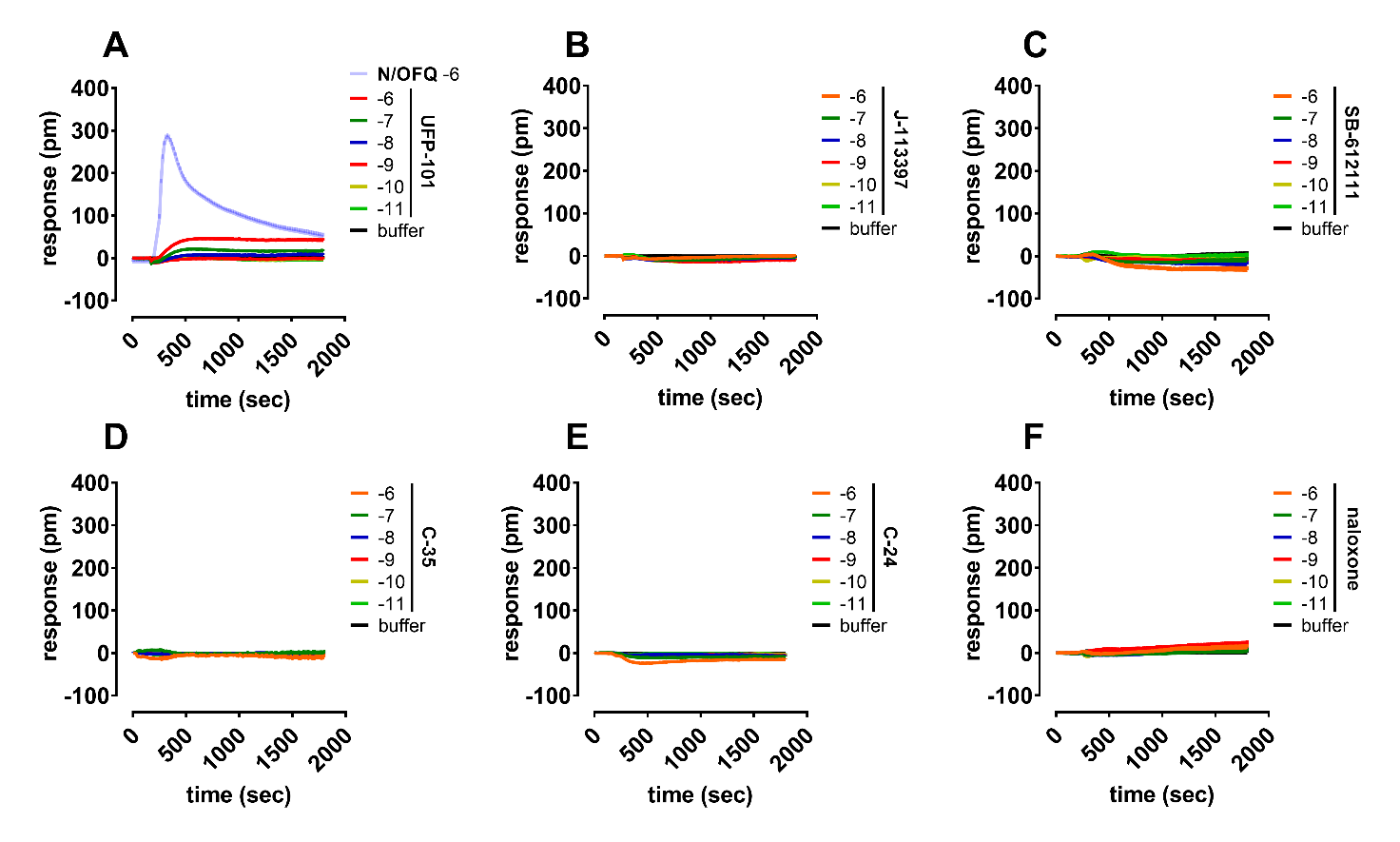

Supplement: S1 Fig — Concentration response curves to UFP-101 (panel A), J-113397 (panel B), SB-612111 (panel C), C-35 (panel D), C-24 (panel E), and naloxone (panel F). Representative traces were obtained from a representative experiment performed in triplicate. (DOCX) [file pone.0203021.s001.docx]
